# Metastatic Endometrial Cancer to the Sigmoid Colon Masquerading as Primary Colorectal Cancer

**DOI:** 10.7759/cureus.19646

**Published:** 2021-11-16

**Authors:** Elliott Koury, Hani Kawar, Elie Chahla

**Affiliations:** 1 Internal Medicine, St. Luke's Hospital, Chesterfield, USA; 2 Gastroenterology and Hepatology, St. Luke's Hospital, Chesterfield, USA

**Keywords:** gi malignancy, unusual sites, metastasis to the colon, endometrial carcinoma, colorectal cancer

## Abstract

A 67-year-old female presented with a chief complaint of hematochezia. Medical history was notable for stage 1a endometrial carcinoma status post treatment with radiotherapy alone. The patient was not considered a candidate for surgical intervention at the time due to multiple underlying comorbidities. Colonoscopy revealed a 4 cm, nonobstructive, friable, and ulcerated mass in the sigmoid colon. Initially this was concerning for a primary colorectal carcinoma, although immunohistochemistry revealed a uterine endometrial origin of the lesion. A total hysterectomy was eventually performed along with surgical resection of the affected segment of the colon, which was followed by radiation therapy.

This case illustrates an unusual site of metastasis for endometrial carcinoma. The colonic metastasis of endometrial adenocarcinoma is reported to be rare and unusual, especially in the absence of endometriosis. Immunohistochemistry staining is an important adjunct in distinguishing the diagnosis of endometrial adenocarcinoma from primary colorectal carcinomas. Primary colon cancers are cytokeratin-7 negative and cytokeratin-20 positive, whereas endometrial cancers are cytokeratin-7 positive and cytokeratin-20 negative. This case is important given the scarcity and peculiarity of metastatic colon cancer originating from uterine adenocarcinomas. The possibility of metastatic disease should be maintained with identification of solitary colonic lesions, especially when there is a prior history of malignancy.

## Introduction

Endometrial cancer is the fourth most common cancer affecting women in the United States and is ranked sixth in terms of cancer-related deaths among women [1]. Colon cancer is the third most common cancer among men and women and is the third leading cause of cancer-related deaths in the United States [1]. Most colon cancers are primary tumors although secondary tumors can be found. These often originate in the lung, breast, or ovaries [2]. Even more uncommon are tumors of the uterine endometrium that metastasize to the colon. These tumors most often occur in the setting of underlying endometriosis [3]. In this case, we present a patient with a colonic mass found to be a metastatic lesion of uterine endometrial primary.

## Case presentation

A 67-year-old Caucasian female presented to our hospital with a chief complaint of persistent bright red blood per rectum. Her medical history was significant for hypertension, hyperlipidemia, diabetes mellitus type 2, coronary artery disease with three prior myocardial infarctions, recurrent cerebrovascular accidents requiring anticoagulation with warfarin, gastroesophageal reflux disease, asthma, and endometrial cancer status post radiation therapy. Fifteen months prior to the current presentation, the patient was noted to have a grade 1 endometrial adenoma but was not considered a good surgical candidate due to multiple comorbidities. Vaginal hysterectomy was considered but due to her long and narrow vagina, this option was deferred initially. Her only treatment option was radiation therapy and brachytherapy. She eventually underwent total abdominal hysterectomy with bilateral salpingo-oopherectomy due to continued pelvic pain. The patient denied any prior history of gastrointestinal (GI) bleeding. Her bleeding was described as one large episode of bright red blood per rectum associated with blood clots. She denied any abdominal pain, nausea, vomiting, diarrhea, constipation, or melena. The most recent colonoscopy was performed four months ago and revealed three diminutive polyps in the transverse colon with pathology confirming tubular adenoma.

**Figure 1:**
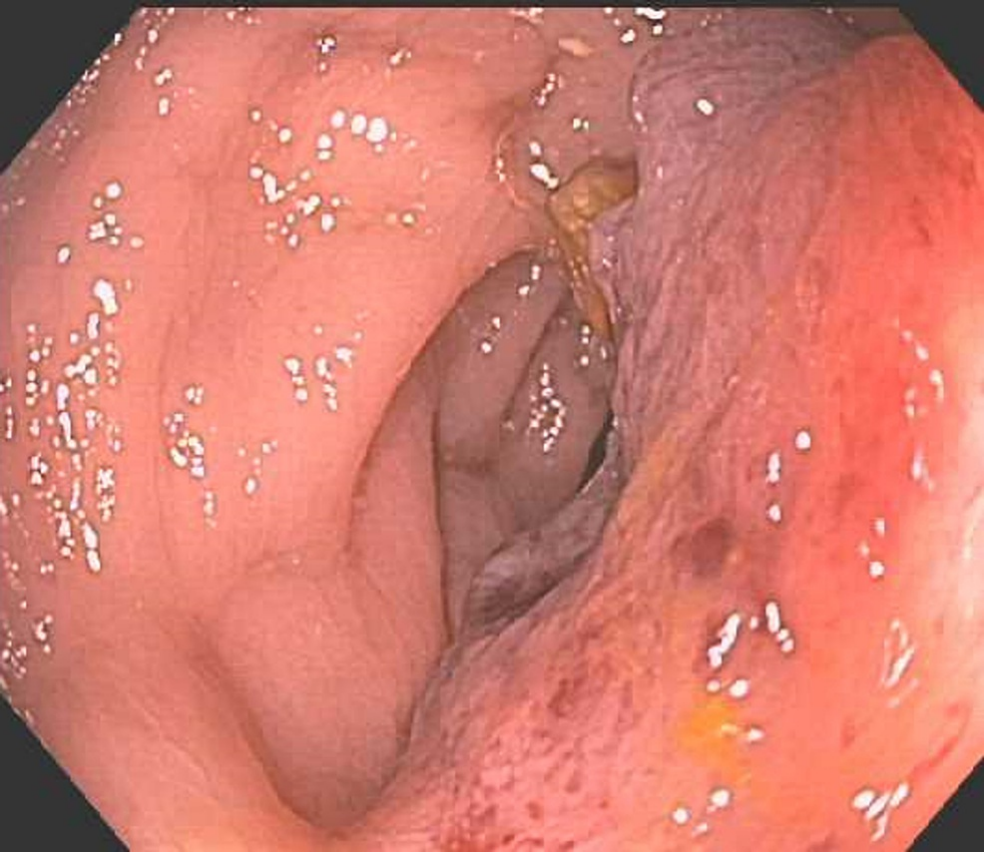
Noncircumferential, fungating, and ulcerated mass in the sigmoid colon.

**Figure 2:**
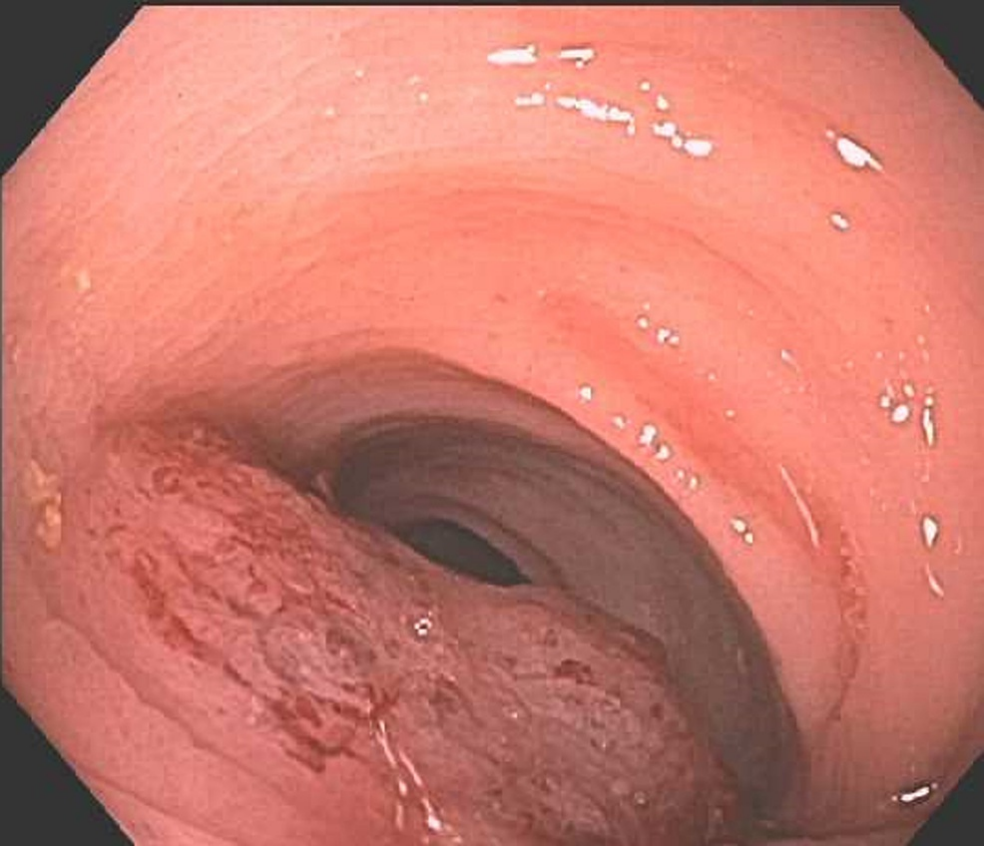
Sigmoid colon mass seen from another angle.

Her physical examination was significant for mild left-sided abdominal tenderness but was otherwise unremarkable. Rectal examination was notable for nonbleeding hemorrhoids and no visible blood. Blood work revealed white blood cells of 14.3k/uL (normal range 4.3-10.0 k/uL) and hemoglobin of 9.6 g/dL (normal range 11.8-14.8 g/dL), which is similar to the patient’s baseline. Creatinine was slightly elevated to 1.2 and blood urea nitrogen was elevated to 39. International normalized ratio was 2.0. Due the large volume of hematochezia and presence of anemia, the patient was admitted to the hospital and underwent a colonoscopy, which revealed a large, fungating, friable, and ulcerated nonobstructing mass in the sigmoid colon. The mass was noncircumferential, measured 4 cm in length, and was located 15-19 cm from the anal verge (Figures 1, 2). Biopsies were obtained with cold forceps for histology and the proximal and distal margins of the mass were tattooed. Histology showed invasive and moderately differentiated carcinoma without visible goblet cells. Given the patient’s history of endometrial cancer, immunohistochemistry was performed and was consistent with an endometrial (endometrioid subtype) primary.

Pan-computed tomography was negative for any obvious distant disease. The tumor marker carcinoembryonic antigen level was normal as well. The patient subsequently underwent surgical resection of the affected colon followed by radiation therapy. At a follow-up visit one year later, the patient was doing well without active GI complaints.

## Discussion

Uterine and colon cancers have been shown to be linked in the medical literature, with a small part of this being attributed to hereditary nonpolyposis colon cancer. This connection is so pervasive that the National Comprehensive Cancer Network guidelines have recommended that women with endometrial or ovarian cancer diagnosed prior to the age of 60 be screened for colorectal cancer at age 40 or at the time of initial diagnosis of the gynecologic tumor [4].

Stage 1 endometrial cancer is usually treated with surgical resection and when contraindicated, radiation therapy is considered [5]. Common sites of metastatic endometrial cancer can involve most commonly the lymph nodes (48%), followed by the vagina, peritoneum, and lung. The colon is an uncommon organ to be involved with this disease. When this does occur, it is most commonly in the setting of endometriosis [3].

Only a handful of cases reported in the medical literature describe endometrial cancer with metastasis to the colon. Anstadt et al. have reported a similar case in which the colonic lesion resembles a primary colon cancer and is only distinguishable by pathology and immunohistochemical analysis [3]. A few other cases have been reported though endometrial malignancies that metastasize to the colon appear to be less commonly reported than primary colon cancers that spread to the cervix and uterus. In addition, atypical sites of endometrial cancer metastasis commonly occur in the setting of and with a background of endometriosis [3]. Our patient did not have a history or current evidence of endometriosis and the mass resembled a primary colonic malignancy. Of the reported cases of endometrial carcinoma with colonic metastasis, very few are in the absence of endometriosis [3]. Immunohistochemistry, especially detection of cytokeratin-7 and cytokeratin-20, is useful in distinguishing endometrial adenocarcinomas from primary colonic tumors. Primary colon cancer is usually cytokeratin-7 negative and cytokeratin-20 positive., whereas endometrial cancers are always cytokeratin-7 positive and cytokeratin-20 negative [2,6].

## Conclusions

Endometrial primary malignancy can metastasize most commonly to the vagina or perineum. Rarely these tumors metastasize to the colon, and if this occurs, it is usually in the setting of endometriosis. Here we have reported the case of a woman with a history of early-stage endometrial cancer treated with radiation therapy alone due to lack of surgical candidacy. She eventually presented with lower GI bleeding a few months after her initial diagnosis and was found to have metastatic disease to the colon and not a primary colon cancer despite the lack of endometriosis. Though rare, endometrial cancer with colonic metastasis should be suspected in patients with known history of endometrial cancer and apparent colon cancer.
